# Superheterodyne-inspired waveguide-integrated metasurfaces for flexible free-space light manipulation

**DOI:** 10.1515/nanoph-2022-0352

**Published:** 2022-09-06

**Authors:** Geng-Bo Wu, Shu-Yan Zhu, Stella W. Pang, Chi Hou Chan

**Affiliations:** State Key Laboratory of Terahertz and Millimeter Wave, City University of Hong Kong, Hong Kong 999077, China; Department of Electrical Engineering, City University of Hong Kong, Hong Kong 999077, China; School of Microelectronics Science and Technology, Sun Yat-sen University, Zhuhai 519000, China; Centre for Biosystems, Neuroscience, and Nanotechnology, City University of Hong Kong, Hong Kong 999077, China

**Keywords:** guided wave, metasurface, photonics, radio communications, superheterodyne

## Abstract

Metasurfaces have attracted significant attention in recent years due to their unprecedented light-manipulation abilities. However, most metasurfaces so far have relied on external light excitation, prohibiting them from full on-chip integration. Inspired by the superheterodyne principle in radio communications, here we propose a new waveguide-integrated metasurface architecture capable of converting in-plane guided modes into any desired out-of-plane free-space modes. A theoretical model, verified by simulation and experiment, is developed to provide a deep understanding of the involved physical mechanism and facilitate innovative metasurface designs. The judicious design of baseband signals allows the silicon-based superheterodyne metasurfaces to achieve complex light manipulations, including arbitrary-direction beam deflection and focusing. The proposed superheterodyne metasurface is a marriage of radio communications and photonics. It provides a paradigm shift of metasurface designs and empowers integrated photonic devices with extraordinary free-space interactivity capability, enabling a broad spectrum of applications in communications, remoting sensing, and imaging.

## 1 Introduction

The exponential growth of data traffic due to emerging applications such as cloud services, augmented/virtual reality, and the Internet of Everything will exhaust the electronic chips’ available transmission and storage capacity, which have nearly reached their integration limit. Like its electronic counterpart, the photonic integrated circuits (PIC) [1–3] integrate multiple photonic components such as waveguides, lasers, polarizers, optical multiplexers/demultiplexers, and phase shifters into a single chip. Driven by photons rather than electrons, PICs feature distinctive advantages such as higher speed, larger integration capacity, lower thermal effect, and lower power consumption. Therefore, PICs provide a viable solution to overcome electronic chips’ integration and heat generation problems. In many applications, such as Li-Fi and LiDAR systems, PICs require an advanced interface that can effectively bridge the guided modes inside the integrated devices and free-space waves to access free space. However, the functionalities of conventional interfaces based on edge coupler [4, 5] and coupling grating [6–8] are extremely limited, and they suffer from high-order diffractions. Coupling light from free space to PICs has been an open challenge at the device level, especially for the high refractive index contrast waveguides.

Recently, metasurfaces have received considerable attention in science and engineering communities due to their extraordinary light manipulation ability. Metasurfaces consist of a planar array of subwavelength structures with spatially varying geometric parameters to engineer the light properties, including phase front, amplitude, polarization, and spectrum [9–13]. However, most studies have hitherto focused on free-space-only or guided-wave-only light control. The theory and methodologies of these metasurfaces are well-explored and mature after years of research and development. Free-space-only metasurfaces use abrupt phase gradient to realize various intriguing phenomena, such as anomalous deflection [14–16], achromatic focusing [17–19], holography [20, 21], and high-resolution imaging [22–24]. However, the input and output of free-space-only metasurfaces are both free-space waves. They require external free-space light excitation in either reflection or transmission modes, prohibiting them from further integrating with light sources within the same chip. On the other hand, guided-wave-only metasurfaces explore the strong optical scattering at the subwavelength intervals to realize diverse functionalities, such as waveguide mode conversion [25], second harmonic generation [26], and unidirectional transmission of guided waves [27]. For guided-wave-only metasurfaces, the input and output waves are both guided waves, and again they cannot realize the guided mode to free-space mode conversion that is highly sought-after for PICs.

Gradient metasurfaces have been proven to behave as a bridge linking guided and free-space modes [28]. Different types of transformation, including guided to free-space waves [29–34] and free-space to guided waves [28, 35, 36], have been reported. A 2*π* phase shift is required for the gradient metasurfaces to compensate for the momentum mismatch between the guided wave and free-space wave. Moreover, these waveguide-integrated metasurfaces generally require additional meta-atoms to couple energy into free space, inevitably increasing the loss and fabrication complexity of the system. The existence of the metallic materials also causes significant optical losses, limiting their application areas.

Signal modulation has been the cornerstone of modern radio communications since the date of Marconi. The purpose of modulation in a communication link is to shift the frequency of the baseband signal (such as audio, image, and video) into other frequencies suitable for transmission [37, 38]. Superheterodyne architecture has been widely popular in modern radio communication systems to perform signal modulation and demodulation [37, 38]. Inspired by the superheterodyne principle in radio communications, we propose a new metasurface architecture that can effectively convert in-plane guided mode into desired out-of-plane free-space mode at will. Our superheterodyne metasurfaces exhibit distinct advantages compared to previously reported waveguide-integrated metasurfaces [25, 39], [40], [41], [42], [43], [44], [45], [46], [47]. First, our superheterodyne metasurfaces are free of the 2*π* phase-shift range requirement for the meta-atoms that is difficult to achieve for waveguide-fed metasurfaces [25, 39], [40], [41], [42], [43], [44], [45], [46], [47]. This is enabled by our proposed spatial superheterodyne architecture that tailors the local spatial frequency of the guided wave to perform spatial frequency modulation (FM). Such a scheme makes our superheterodyne metasurfaces operate entirely differently from other waveguide-integrated metasurfaces. Second, the superheterodyne metasurfaces can effectively control the extracted free-space waves by designing the baseband signals judiciously. We present several examples to illustrate the remarkable light manipulation capability of the superheterodyne metasurfaces. Third, the realization of superheterodyne metasurfaces does not require adding any meta-atoms above the waveguide. We tailor the geometric structure of the waveguide itself by etching the 2D lattice of air holes in the silicon (Si) waveguide to engineer the local spatial frequency. The dielectric-based design introduces negligible insertion losses to PICs and hence has higher efficiency. Finally, our superheterodyne metasurfaces can be fabricated with high accuracy using lithography steps similar to conventional photonic waveguides made by the well-established semiconductor processing technology, making their integration with PICs straightforward. The superheterodyne-inspired metasurface is an excellent example showing how two seemingly unrelated fields of science can be combined, opening up new ways of metasurface design.

## 2 Results

### 2.1 Theoretical formulation

**Figure 1:**
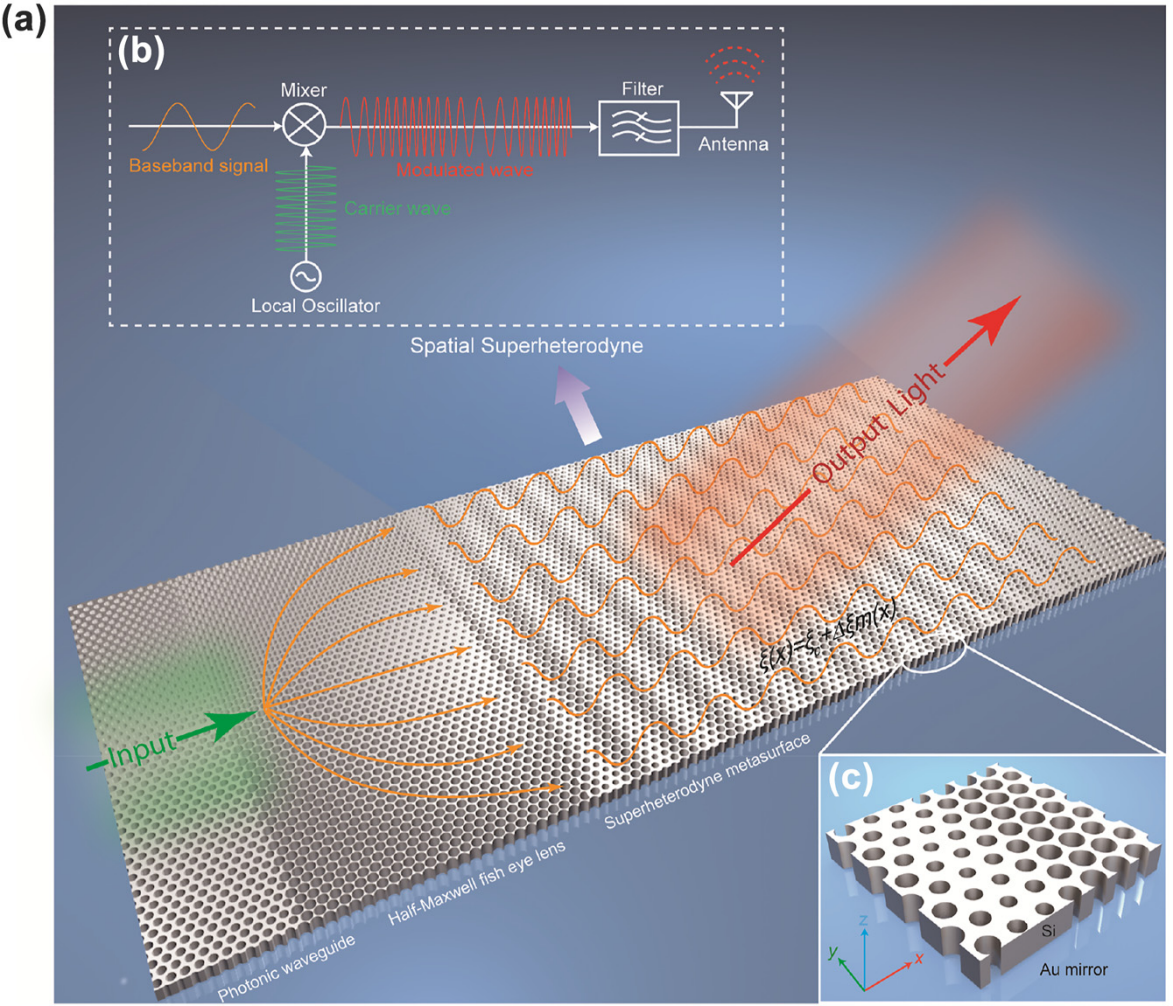
Conceptual illustration of the waveguide-integrated superheterodyne metasurfaces for free-space coupling. (a) Configurations of the whole metasurface, which consists of a photonic waveguide, planar half-Maxwell fish eye lens and superheterodyne metasurface – all implemented in a Si slab with air-hole cladding. The photonic waveguide and the planar half-Maxwell fish eye lens are utilized to guide and generate collimated guided waves to feed the superheterodyne metasurface. The superheterodyne metasurface can convert and mold the in-plane guided modes into any desired out-of-plane free-space modes. (b) The functionality of the metasurface can be interpreted as a spatial superheterodyne transmitter to perform spatial frequency modulation of the guided waves. New spatial harmonics are generated, but only the target spatial spectra, corresponding to the desired free-space modes, are selected by an equivalent spatial filter by the momentum matching condition and eventually radiate into free space. (c) The metasurface is implemented in the form of a 2D lattice of sub-wavelength air hole structure in a Si slab. An Au ground works as a mirror and provides mechanical support to the THz metasurface.

A conceptual illustration of the superheterodyne metasurface based on the Si platform is shown in Figure 1(a). The photonic waveguide and the planar half-Maxwell fish eye lens are utilized to guide and generate the planar wavefront to feed the superheterodyne metasurface, respectively. The concept of the superheterodyne metasurface borrows the superheterodyne principle from radio communications, as illustrated in Figure 1(b). But different from traditional radio superheterodyne transmitters temporally modulating the signal, our superheterodyne metasurfaces operate in the spatial domain. The superheterodyne metasurfaces modulate the spatial carrier wave according to the baseband signal. The process of light conversion from guided to free-space modes is akin to a superheterodyne transmitter to launch radio into free space but in the spatial domain.

**Figure 2:**
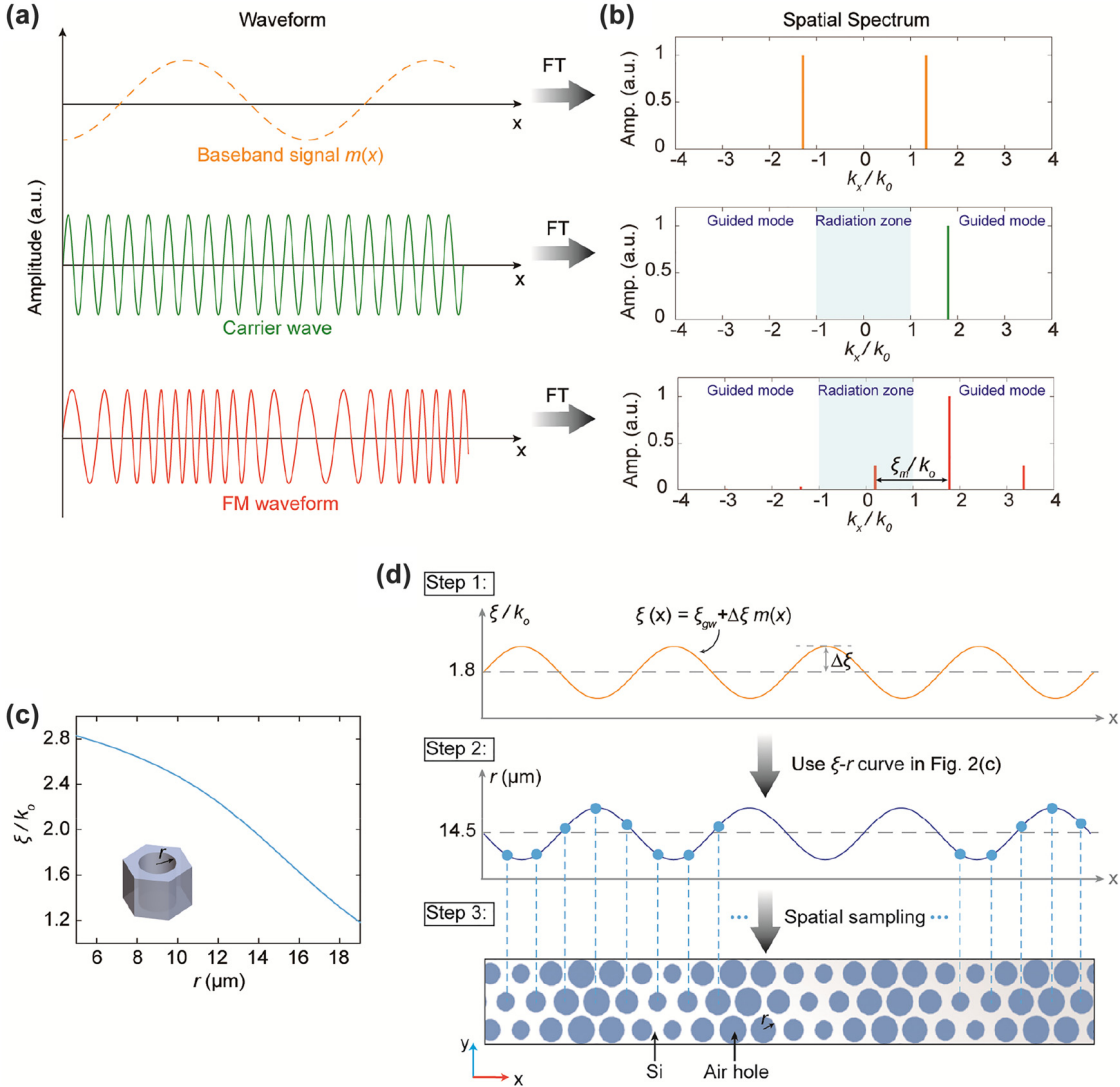
Working principle of the superheterodyne metasurfaces. (a) Waveforms of the baseband signal, carrier wave, and the modulated wave after the spatial frequency modulation. The carrier wave corresponds to the guided wave inside waveguides with uniform air holes. The dashed line of the baseband signal indicates it is fictitious, not representing any real waveform in the Si substrate. (b) The corresponding spatial spectra after performing spatial Fourier transform of the waveforms in (a). (c) Simulated local spatial frequency as a function of the radius of the air hole at 1 THz. Inset shows the configurations of the unit cell. (d). The detailed method of modulating the carrier wave. The radius of the air hole at each coordinate is selected to fulfill the required local spatial frequency pertinent to the baseband information.

To clearly illustrate the concept, we first consider a Si waveguide with uniform air holes with a radius of 14.5 μm, and the corresponding wavenumber is *ξ*_gw_ = 1.8*k*_0_ (*k*_0_ is the free-space wavenumber at 1 THz). The uniform waveguide without any spatial modulation means Si substrate with uniform air holes. The wavenumber of the guided wave *ξ*_gw_ can be determined using the *ξ*-*r* curve in Figure 2(c). We denote the unmodulated guided wave in this uniform waveguide as the carrier wave herein, whose waveform is illustrated in Figure 2(a) in green color. The electric field distribution of the carrier wave is expressed as $E\left(r,t\right)=E_{0}{\text{e}}^{\text{i}\xi_{\text{gw}}x}{\text{e}}^{-\text{i}\omega t}\delta \left(z\right)\hat{x}$. Since the wavenumber of the guided wave is larger than that in free space, i.e., *ξ*_gw_ > *k*_0_, the carrier wave is bounded to the waveguide and cannot be radiated into free space due to the momentum mismatching with free space.

It is known that FM in radio communications is characterized by varying the instantaneous frequency of the carrier wave in accordance with the time-variant baseband signal [37, 38]. Here we perform the spatial FM, described as the local spatial frequency *ξ* of the carrier wave modulated by the spatial-variant baseband signal *m*(*x*)

(1) $$\xi \left(x\right)≜\xi_{\text{gw}}+\Delta \xi m\left(x\right)$$

In analogy to the instantaneous frequency in FM radio, the local spatial frequency is given as $\xi =\frac{\partial \phi }{\partial x}$, where *ϕ* is the phase of the wave at coordinate *x*. In Eq. (1), *ξ*_gw_ is the spatial frequency of the unmodulated carrier wave. Δ*ξ* is the spatial frequency deviation, indicating the maximum shift of *ξ*(*x*) relative to the carrier spatial frequency *ξ*_gw_. The spatial FM waveform can be written as

(2) $$E\left(r,t\right)=E_{0}{\text{e}}^{\text{i}\int^{x}\xi \left(x^{\prime }\right)dx^{\prime }}{\text{e}}^{-\text{i}\omega t}\delta \left(z\right)\hat{x}$$

For the simplest single-tone spatial FM scenario, i.e., the baseband signal is $m\left(x\right)=\cos\left(\xi_{m}x\right)$, the spatial FM waveform is simplified as

(3) $$E\left(r,t\right)=E_{0}{\text{e}}^{\text{i}\left[\xi_{\text{gw}}x+M\text{sin}\left(\xi_{m}x\right)\right]}{\text{e}}^{-\text{i}\omega t}\delta \left(z\right)\hat{x}$$

where $M=\frac{\Delta \xi }{\xi_{m}}$ is defined as modulation index. After performing the single-tone spatial FM, the modulated waveform is illustrated in Figure 2(a) in red color. Based on the field distribution along the waveguide aperture in Eq. (3), the radiation field above the waveguide can be obtained by solving the Maxwell equations, and the exact solution is given as (see Supplementary Note 1 for the detailed derivations)

(4) $$\begin{array}{ll} E\left(r,t\right) & =E_{0}\overset{\infty }{\underset{n=-\infty }{\sum }}\left(\hat{x}-\frac{\xi_{\text{gw}}+n\xi_{m}}{k_{zn}}\hat{z}\right)\\ & \;\times J_{n}\left(M\right){\text{e}}^{\text{i}k_{zn}z}{\text{e}}^{\text{i}\left(\xi_{\text{gw}}+n\xi_{m}\right)x}{\text{e}}^{-\text{i}\omega t} \end{array}$$

in the region *z* > 0. In Eq. (4), *J*_*n*_(*M*) is the *n*th order Bessel function of the first kind with the argument *M*. We can observe from Eq. (4) that an infinite number of space harmonics are generated for the spatial FM waveform.

Harmonic analysis based on the Fourier transform has been proven powerful in describing and analyzing signals and systems in radio communications; this inspires us to leverage space harmonic analysis to investigate the spatial FM. Figure 2(b) depicts the spectrum distributions of the baseband, carrier, and the modulated waves by taking spatial Fourier transform of the waveforms in Figure 2(a) (See Supplementary Note 2 for the detailed mathematical expression). In analogy with the spectrum of FM radio, the spatial FM waveform consists of a carrier component and an infinite set of spatial frequencies located on the two sides of the carrier with a separation of *ξ*_*m*_. It is well known that there is a one-to-one correspondence between the free-space waves and the spatial harmonics provided that $-1<\frac{k_{x}}{k_{0}}<1$. From Figure 2(b), we can observe that the spatial FM waveform has spatial frequency components that lie within the radiation zone $\left(-1<\frac{k_{x}}{k_{0}}<1\right)$, making the guided to free-space mode conversion possible. Different from the FM in radio communications, the baseband signal in the spatial superheterodyne metasurface is fictitious for indicating how the metasurface is modulated, not representing any real waveform in the Si substrate. The FM waveform (red color) in Figure 2(a) represents the real waveform propagating inside the superheterodyne metasurface, which supports an infinite number of diffraction modes. As illustrated by the spatial frequency distribution of the FM waveform shown in Figure 2(b), only the lower spatial harmonic frequency is fallen into the radiation zone and radiates into free space, while other spatial harmonics are slow waves and bounded to the metasurface. Therefore, the wave continues to propagate along the metasurface while radiating into free space. According to the FM in radio communication theory [37, 38], modulation efficiency is defined as the percentage of the total power of the modulated signal that conveys information. For the spatial superheterodyne metasurface, only the first lower spatial frequency component is in the visible region $\left(-1<\frac{k_{x}}{k_{0}}<1\right)$ that radiates into free space. Therefore, the modulation efficiency is given by $\eta_{m}=\frac{|J_{-1}\left(M\right){|}^{2}}{\sum_{n=-\infty }^{+\infty }|J_{n}\left(M\right){|}^{2}}$. For the modulation index *M* = 0.25 in this design, the modulation efficiency is 1.54%. It is important to point out that the superheterodyne metasurface is deliberately designed to have a low modulation efficiency. Consequently, each meta-atom only radiates a small portion of the power from the Si substrate into free space, and the remaining of the power is further delivered to the rest of meta-atoms. In this manner, the meta-atoms downstream can also have enough excitation to form a large radiating aperture of the metasurface.

The purpose of conducting spatial single-tone FM is to introduce a tangential momentum *ξ*_*x*_ to the original momentum of the guided wave such that it can match the momentum of free space. This distinguishes the superheterodyne metasurfaces from traditional waveguide-integrated metasurfaces, which require at least a 2*π* phase-shifting range by the meta-atoms to compensate for the phase accumulation from the propagation of the guided wave [40]. The magnitude of the imparted tangential momentum by the spatial FM equals to the spatial frequency of the baseband signal, i.e., *ξ*_*x*_ = −*ξ*_*m*_. The output angle of the extracted optical wave for the superheterodyne metasurface is given as

(5) $$\theta_{r}=\text{si}{\text{n}}^{-1}\frac{1}{k_{0}}\left(\xi_{\text{gw}}-\xi_{m}\right)$$

Due to the finite length of the metasurface aperture, the truncation effect results in multiplying a window function $\Pi \left(x\right)=\left\{\begin{array}{c} 1,\;\;-\frac{L}{2}<x<\frac{L}{2}\;\\ 0,\;\;\;\;\;\;\;\;\;\;\;\;\;\;\;\;else\; \end{array}\right.$ to the original field distribution, corresponding to a convolution in the spectral frequency domain. Considering the first lower spatial frequency harmonic to be radiated, the scattering pattern of the superheterodyne metasurface can be written as

(6) $$\begin{array}{c} F\left(\theta \right)=FT\left\{E_{0}J_{-1}\left(M\right){\text{e}}^{\text{i}\left(\xi_{\text{gw}}-\xi_{m}\right)x}\cdot \Pi \left(x\right)\right\}\\ =\frac{E_{0}J_{-1}\left(M\right)\sin\left\{\frac{\left[k_{0}\sin\theta -\left(\xi_{\text{gw}}-\xi_{m}\right)\right]L}{2}\right\}}{\pi \left[k_{0}\sin\theta -\left(\xi_{\text{gw}}-\xi_{m}\right)\right]} \end{array}$$

where *θ* is the angle measured from the broadside direction, and *L* is the length of the metasurface along the *x*-direction. The truncation effect will generate sidelobes, and a smaller truncation length *L* results in a larger half-power beamwidth (See Supplementary Note 9 for the detailed effects).

In a physical sense, our waveguide-integrated metasurface can be interpreted as a spatial superheterodyne transmitter to implement spatial FM, as shown in Figure 1(b). The unmodulated carrier wave is treated as a local oscillator to launch a single-tone waveform. First, the metasurface works as a spatial frequency mixer for combining the baseband and the carrier signals. New spatial frequencies are generated after the spatial frequency mixer. The spatial frequencies within the radiation zone correspond to out-of-plane propagating modes. The rest are evanescent modes bounded to and propagating along the waveguide. This behavior is equivalent to an ideal band-pass spatial filter invariably passing a finite block of spatial frequencies while completely removing those outside the passband. Due to the momentum matching, those spatial frequencies passing through the spatial filter can be radiated into free space, equivalent to an antenna transmitting radio waves into space. A single superheterodyne metasurface realizes all the above functionalities, including mixing, filtering, and radiating, as in a conventional superheterodyne system. Since the proposed waveguided-fed metasurface can transmit electromagnetic (EM) waves like conventional antennas, the superheterodyne metasurface can also transmit time-domain information if the input EM waves are temporally modulated by baseband signals.

The conventional superheterodyne transmitter in radio communications is in the time domain, and the carried information (such as audio and video) in the baseband signal is also time-domain information. The superheterodyne metasurface mimics the superheterodyne architecture yet in the spatial domain. Therefore, the superheterodyne metasurface can carry spatial-domain information. For example, when the baseband signal is a sinusoidal wave, it carries the spatial information of the generated free-space wave, including the beam type (i.e., high-directivity pencil beam in the far field) and the main beam direction. For the light focusing application, the baseband signal in the superheterodyne metasurface carries the spatial information of the generated focal point position. Since the baseband signal of the superheterodyne metasurface carries the spatial information of the generated waves, one can flexibly manipulate the generated free-space light by judiciously designing the baseband signal. It should be reiterated that the superheterodyne approach is adopted to design a metasurface antenna with specific radiation beam characteristics, not modulating a baseband signal onto a carrier frequency. In what follows, we exemplify our superheterodyne metasurfaces with powerful light controllability by demonstrating out-of-plane beam deflection, focusing, and 2D arbitrary direction deflection.

### 2.2 Out-of-plane deflection

As a proof of concept, the superheterodyne metasurface is implemented in a Si-slab platform operating at 1 THz. To engineer the local spatial frequency of light in the waveguide, we consider a 2D lattice of densely packed, subwavelength air holes in the Si slab, as shown in Figure 1(c). An Au ground acts as a mirror for the field and provides mechanical support to the metasurface at the THz spectrum. Since the period of the air-hole is much smaller than the wavelength of the light, the diffraction effect can be suppressed, and the structure behaves as an effective bulk homogeneous material. The local spatial frequency of the Si slab is determined by the volumetric fill factor of the air inclusions. For the fundamental TM_01_ mode in the Si-slab waveguide, the relationship between the local spatial frequency and the air-hole radius is given in Figure 2(c), obtained by a commercially available CST Studio Suite numerical simulator (Detailed simulation setup is provided in the Methods section). The detailed method of modulating the carrier wave is illustrated in Figure 2(d). In the first step, the local spatial frequency *ξ* can be obtained according to Eq. (1) once the baseband signal *m*(*x*) is determined. The second step is to use the *ξ*-*r* matching curve in Figure 2(c) to calculate the corresponding required radii of the air-holes along the Si substrate. Due to the discrete nature of the metasurface, the final step is to spatially sample the radii curve at each unit cell coordinate. According to the Nyquist Sampling Theorem, the spatial sampling should be smaller than half of the wavelength in the waveguide at the operating frequency.

**Figure 3:**
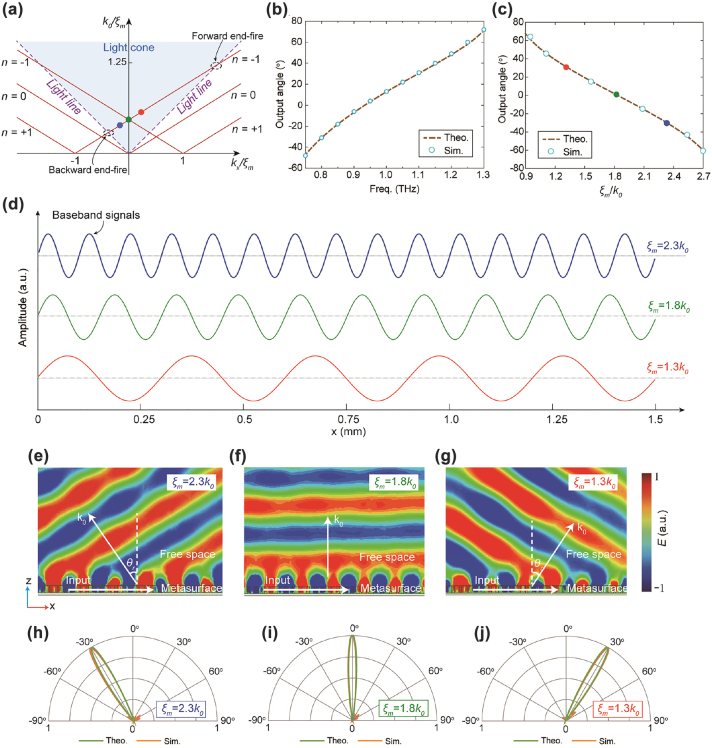
Agile beam deflection. (a) Brillouin diagram of the superheterodyne metasurfaces for agile beam deflection via changing the baseband spatial frequency or operating frequency. (b) Output beam angle versus the operating frequency when the baseband spatial frequency is fixed as *ξ*_*m*_ = 3.34 × 10^4^ rad/m. The dashed line and the dots depict the theoretical and simulated data, respectively. (c) Output beam angle versus the normalized baseband spatial frequency at 1 THz. The dashed line and the dots represent the theoretical and simulated data, respectively. (d) The waveforms of the baseband signals with different spatial frequencies *ξ*_*m*_ = 2.3*k*_0_, 1.8*k*_0_, and 1.3*k*_0_, which corresponds to the blue, green, and red dots in (a) and (c), respectively. (e to g) Simulated electric field distributions of the superheterodyne metasurfaces modulated by different frequencies of the baseband signals *ξ*_*m*_ = 2.3*k*_0_, 1.8*k*_0_, and 1.3*k*_0_ at 1 THz, respectively. (h–j) Theoretical and simulated scattering patterns for the superheterodyne metasurfaces modulated by different frequencies of the baseband signals *ξ*_*m*_ = 2.3*k*_0_, 1.8*k*_0_, and 1.3*k*_0_ at 1 THz, respectively.

Here we demonstrate the superheterodyne metasurface for agile beam deflection through changing the operating frequency or the spatial frequency of the baseband signal. We leverage the Brillouin diagram, a plot of *k*_0_/*ξ*_*m*_ versus *k*_*x*_/*ξ*_*m*_ as illustrated in Figure 3(a), to analyze and interpret the single-tone spatial FM results. The shaded region with the boundaries *k*_0_ = ±*k*_*x*_ indicates the light cone. The first lower spatial frequency lies within the light cone that will be radiated into free space. We observe that the extracted light direction can steer from the backward end-fire to the forward end-fire by varying the value of *k*_0_/*ξ*_*m*_. First, we fixed the spatial frequency of the baseband signal *ξ*_*m*_ = 3.34 × 10^4^ rad/m, while varying the operating frequency (and hence changing the free-space wavenumber *k*_0_) from 0.75 to 1.3 THz. The theoretical output angles versus the operating frequency calculated by Eq. (5) are depicted in the dashed line in Figure 3(b), agreeing well with the simulated results in cyan dots. On the other hand, the output angle can also be flexibly controlled by varying the spatial frequency of the baseband signal *ξ*_*m*_ at the fixed operating frequency, as illustrated in Figure 3(c). We consider three sinusoidal baseband singles with spatial frequencies of *ξ*_*m*_ = 2.3*k*_0_, 1.8*k*_0_, and 1.3*k*_0_, respectively, whose waveforms are depicted in Figure 3(d). The geometric parameters of the air-hole inclusions of the Si slab for each case can be synthesized using the matching curve in Figure 2(c). The simulated electric field distributions for the three radiating cases are illustrated in Figure 3(e)–(g), respectively, from which we clearly observe that the waves can be extracted and molded into the intended directions in free space. Figure 3(h)–(j) compare the theoretical scattering patterns calculated by Eq. (6) with the simulated ones for the three superheterodyne metasurfaces modulated by different baseband signals, respectively. We can observe that the extracted light is pointed to the intended direction of −30°, 0°, and +30°, respectively. The simulated conversion efficiency, defined as the ratio of the radiated power into free space to the accepted power by the metasurface, is 86% for the superheterodyne metasurface. These results demonstrate that our superheterodyne metasurface can translate in-plane guided modes into out-of-plane free-space optical modes, whose output angle can be flexibily controlled by varying the spatial frequency of the baseband signal.

### 2.3 2D arbitrary direction deflection

**Figure 4:**
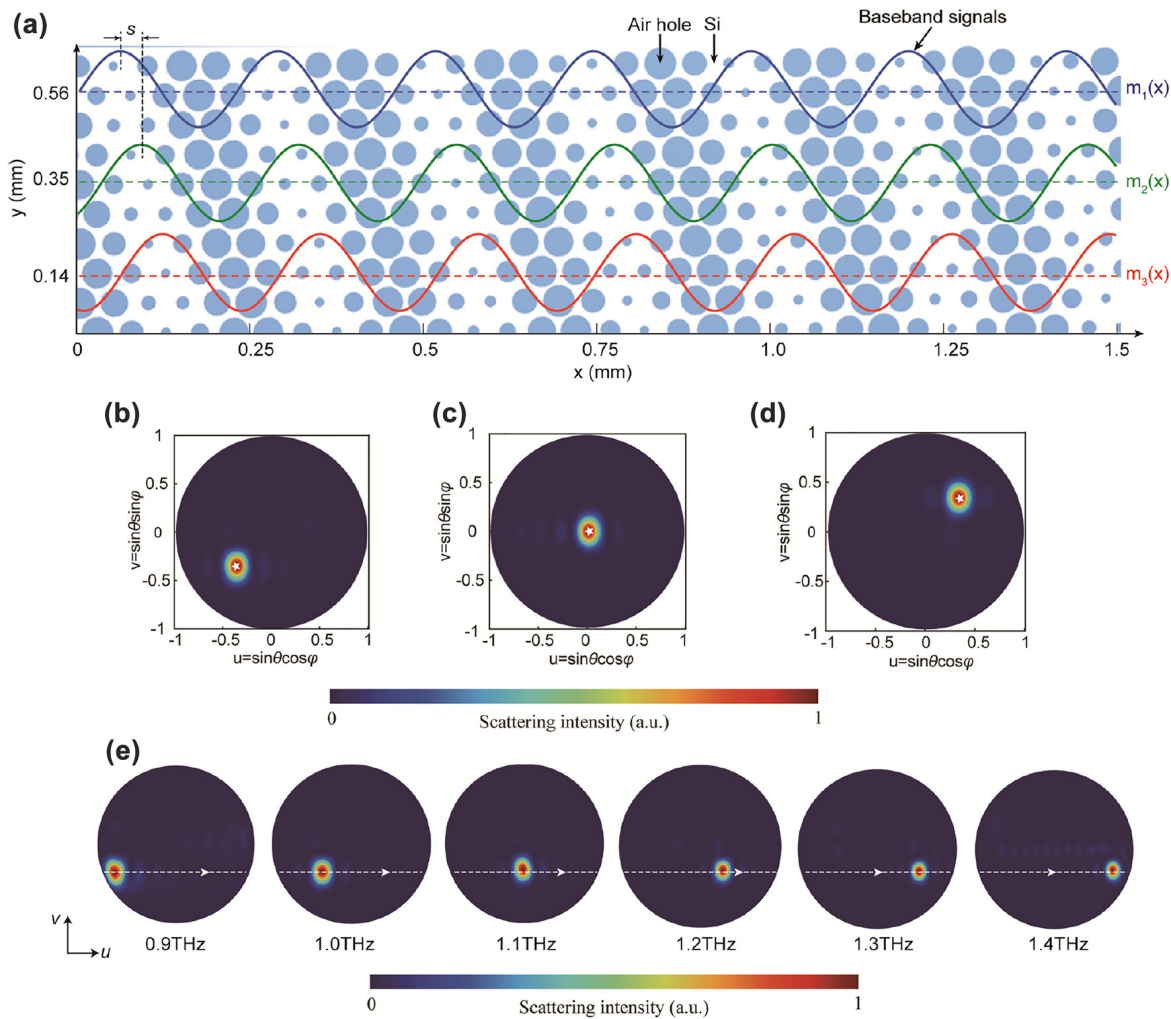
Arbitrary direction deflection. (a) Baseband signal distributions with space shifts *s* along the *y*-direction. The radii of the air-holes are synthesized according to the baseband signals. (b to d) Simulated scattering patterns in *uv*-space at 1 THz by introducing different *x*- and *y*-direction tangential momentums (*ξ*_*x*_, *ξ*_*y*_) = (−1.45*k*_0_, 0.35*k*_0_), (−1.8*k*_0_, 0), and (−2.15*k*_0_, −0.35*k*_0_), respectively. The theoretical output directions are marked as white stars. (e) Simulated scattering patterns in *uv*-space from 0.9 to 1.4 THz with an increment of 0.1 THz. The white dashed line depicts the theoretical frequency-scanning trajectory of the output light.

In the above superheterodyne metasurfaces, only beam deflection in the *xz*-plane is achieved, whereas 2D manipulation of the extracted free-space light has not been shown. Here we demonstrate that superheterodyne metasurfaces can achieve 2D arbitrary direction radiation by further introducing a space translation along the *y*-direction to the baseband signals. First, we consider two sinusoidal baseband signals having an identical spatial frequency but with a space translation *s*, i.e., $m_{2}\left(x\right)=m_{1}\left(x-s\right)$, as depicted in the blue and green lines in Figure 4(a). Suppose that ${\tilde{F}}_{1}$ and ${\tilde{F}}_{2}$ are the spatial frequency spectra of the superheterodyne metasurfaces modulated by the baseband signals *m*_1_(*x*) and *m*_2_(*x*), respectively, then we have the relationship

(7) $${\tilde{F}}_{2}\left(k_{x}\right)={\text{e}}^{\text{i}\left(\xi_{\text{gw}}-k_{x}\right)s}{\tilde{F}}_{1}\left(k_{x}\right)$$

The detailed derivation of Eq. (7) is given in Supplementary Note 3. From Eq. (7), we notice that a space translation of the baseband signal *s* brings an additional phase shift (*ξ*_gw_ − *k*_*x*_)*s* with unchanged amplitude distribution in the spectrum domain. This can be explained by the shift theorem of spatial Fourier transform, which refers to translation in the space domain corresponding to a linear phase shift in the spatial frequency domain. The imparted *y*-direction momentum at the output direction is

(8) $$\begin{array}{c} \xi_{y}=\text{d}\left[\left(\xi_{\text{gw}}-k_{x}\right)s\right]/dy\\ =-\xi_{m}ds/dy \end{array}$$

As a result, we can introduce *x*- and *y*-direction momentums *ξ*_*x*_ and *ξ*_*y*_ to the guided waves. *ξ*_*x*_ is imparted by the spatial FM modulation, while *ξ*_*y*_ is caused by the space shifting. In this manner, the output deflection angle in any 2D direction can be achieved.

Without loss of generality, consider three superheterodyne metasurfaces whose intended free-space light output angles are (*θ*, *φ*) = (30°, 45°), (0°, 0°), and (30°, −135°), respectively. The required tangential momentums are (*ξ*_*x*_, *ξ*_*y*_) = (−1.45*k*_0_, 0.35*k*_0_), (−1.8*k*_0_, 0), and (−2.15*k*_0_, −0.35*k*_0_), respectively. The corresponding simulated 2D scattering patterns in *uv*-space for the three radiating cases are depicted in Figure 4(b)–(d), where the intended output directions are marked as white stars. We clearly observe that the output beam can be correctly pointed to the intended direction. These results verify the 2D free-space light manipulation capability of our superheterodyne metasurface. We also investigate the dispersion properties of the 2D deflection beam. The above section shows that the imparted *x*-direction momentum *ξ*_*x*_ varies with the working frequency (See Figure 3(b)). By contrast, we can observe from Eq. (8) that the imparted *y*-direction momentum *ξ*_*y*_ is frequency independent. As a result, the output direction of the extracted light will steer along the *u*-direction with increasing operating frequency, as marked by the white dashed line in Figure 4(e). The simulated 2D scattering patterns in *uv*-space at different frequencies are also given in Figure 4(e). We clearly see that the beam steers with the frequency in line with the trajectory predicted by the theoretical model.

### 2.4 Focusing flexibility

**Figure 5:**
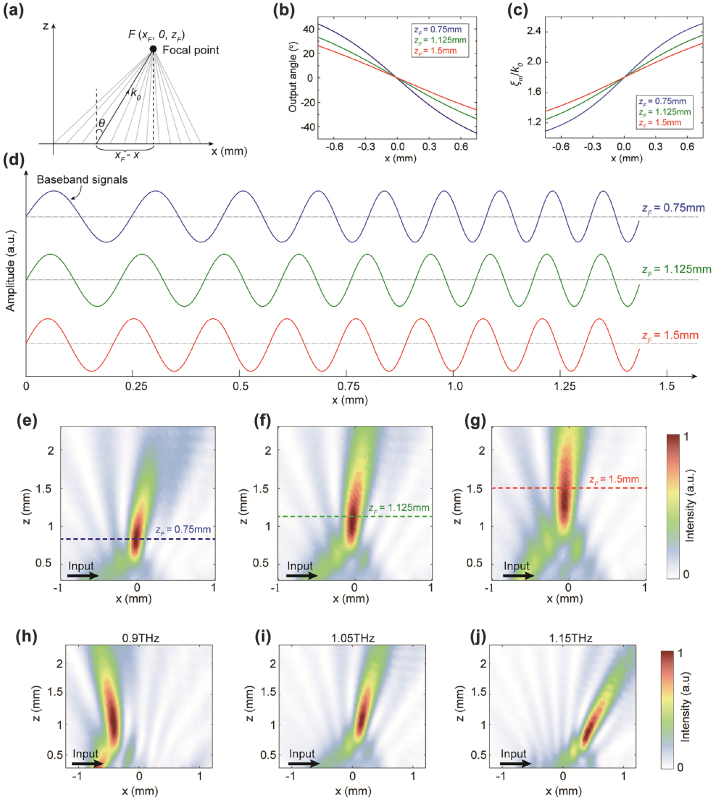
Light focusing flexibility of the superheterodyne metasurface. (a) Geometric representation of the light-focusing metasurface. The superheterodyne metasurface is located at *z* = 0, and the target focal point is at *F*(*x*_*F*_, 0, *z*_*F*_). (b) Calculated output angles of the free-space light along the metasurface aperture when the target foci are *F*(*x*_*F*_, 0, *z*_*F*_) = (0, 0, 0.75 mm), (0, 0, 1.125 mm), and (0, 0, 1.5 mm), respectively. (c) The corresponding spatial frequencies of the baseband signals along the metasurface aperture for different target foci. (d) The corresponding baseband signals for generating different target foci. (e to g) The corresponding simulated intensity distributions for different focusing cases at 1 THz. The dashed lines depict the theoretical vertical levels of the focus point. (h to j) Simulated intensity distributions of the superheterodyne metasurface at 0.9, 1.05, and 1.15 THz, respectively.

In previous sections, the baseband signals are all sinusoidal waves, and accordingly, their output light is high-directivity beams. In analogy with radio communications, where the baseband/message signals can be audio or images, the baseband signals of the superheterodyne metasurfaces are not necessary to be sinusoidal waves such that more complex light manipulation can be achieved. Here we demonstrate that the superheterodyne metasurface can focus the extracted light into a designated focal point *F* = (*x*_*F*_, 0, *z*_*F*_) in free space, as shown in Figure 5(a). Based on the geometric ray approach and the geometrical relationship, the output angle of the free-space light along the superheterodyne metasurface aperture should satisfy $\tan\theta \left(x\right)=\frac{x_{F}-x}{z_{F}}$. As proof-of-concept examples, we consider three focusing cases with the intended focal points *F* = (0, 0, 0.75 mm), (0, 0, 1.125 mm), and (0, 0, 1.5 mm) at 1 THz for superheterodyne metasurfaces with an aperture length of 1.5 mm. The calculated output angles along the metasurface aperture for the three focusing cases are given in Figure 5(b). The corresponding spatial frequencies of the baseband signals along the metasurface aperture according to Eq. (5) are depicted in Figure 5(c). The required baseband signal can be calculated by

(9) $$\begin{array}{c} m\left(x\right)=\cos\left[\overset{x}{\underset{0}{\int }}\xi_{m}\left(x^{\prime }\right)dx^{\prime }\right]\\ =\cos\left[\overset{x}{\underset{0}{\int }}\xi_{\text{gw}}-k_{0}\sin\theta \left(x^{\prime }\right)dx^{\prime }\right]\\ =\cos\left[\xi_{\text{gw}}x-k_{0}\overset{x}{\underset{0}{\int }}\sin\left(\text{ta}{\text{n}}^{-1}\frac{x_{F}-x^{\prime }}{z_{F}}\right)dx^{\prime }\right] \end{array}$$

The synthesized waveforms of the baseband signals are illustrated in Figure 5(d). We can observe that the baseband signals are no longer standard sinusoidal waves; the spatial frequency of the baseband signals increases along the metasurface aperture. The simulated intensity distributions at 1 THz for the three focusing cases are illustrated in Figure 5(e)–(g), respectively, from which we clearly observe that the extracted waves can be concentrated into the intended focal spots. Moreover, we can leverage the dispersion properties of the superheterodyne metasurfaces to achieve frequency focus scanning. Figure 5(h)–(j) illustrate the simulated intensity distributions at 0.9, 1.05, and 1.15 THz, respectively. We can see that the focal spot steers from the backward to the forward directions as the operating frequency increases. These results verify the superheterodyne metasurface in the successful realization of light focusing.

### 2.5 Experimental and characterization results

**Figure 6:**
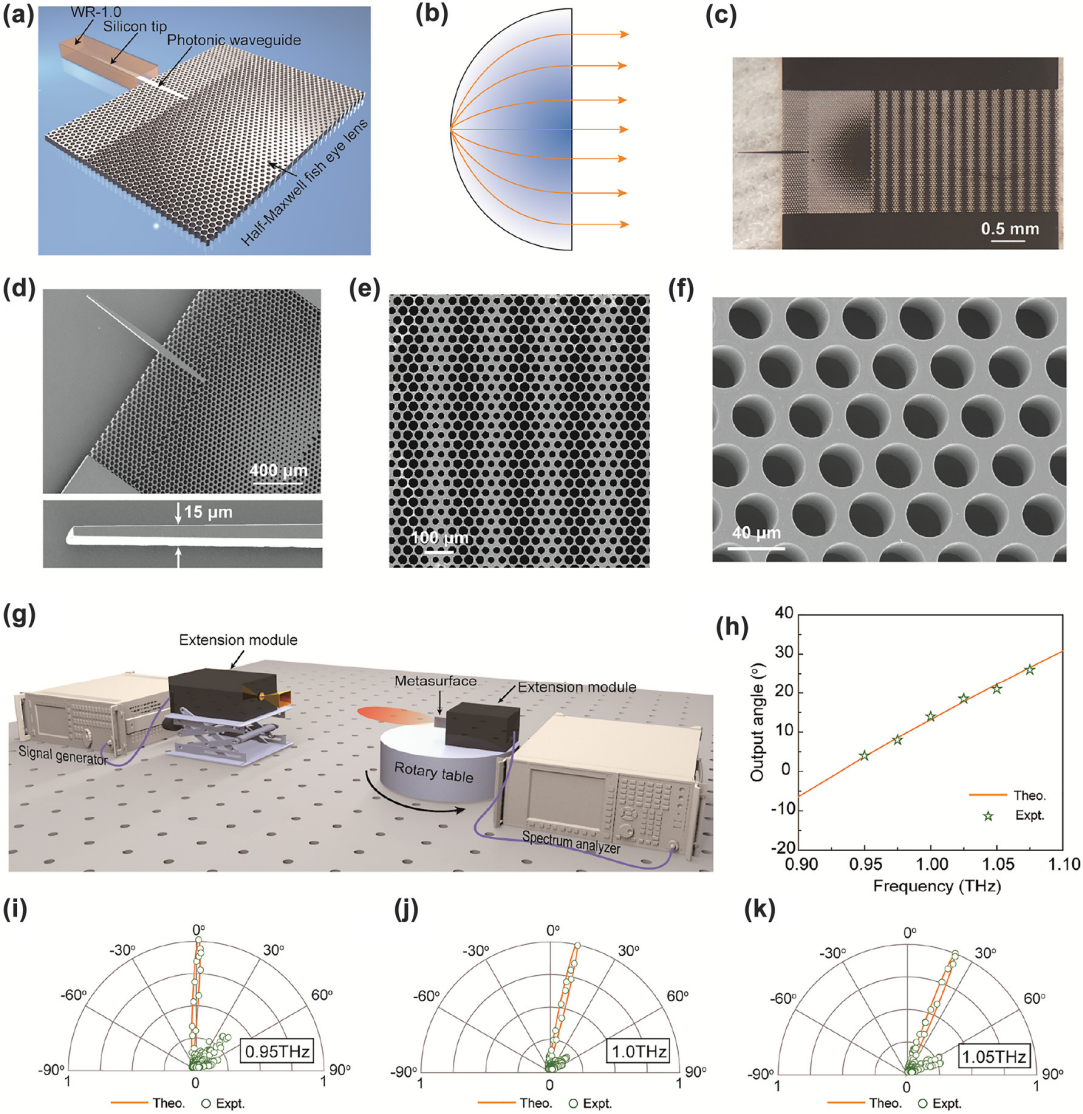
Prototype fabrication and characterization. (a) Schematic of the off-chip measurement of the superheterodyne metasurface. A Si taper tip, a photonic waveguide, and a planar half-Maxwell fisheye lens are utilized to feed the superheterodyne metasurface. The silicon tip is inserted into a standard WR-1.0 waveguide to couple the energy. (b) Working principle of the planar half-Maxwell fish eye lens that generates collimated guided waves to feed the superheterodyne metasurface. (c) Photo of the fabricated prototype. (d to f) Scanning electron micrographs of the silicon tip and half-Maxwell fish eye lens, superheterodyne metasurface, and 2D lattice of air hole of the Si slab, respectively. (g) THz measurement setup to characterize the scattering patterns of the superheterodyne metasurface. (h) Experimental and theoretical output angles of the superheterodyne metasurface at different frequencies. (i to k) Experimental and theoretical scattering patterns at 0.95, 1.0, and 1.05 THz, respectively.

To validate the superheterodyne metasurface concepts and designs, we implement a Si-based superheterodyne metasurface pertaining to the out-of-plane beam deflection example. The fabrication process of the superheterodyne metasurface for focusing is similar to that of the directional radiation. However, the focusing measurement at THz frequencies is much more challenging for our available facilities (See Supplementary Note 6). As a result, only the far-field directional radiation is measured as a proof-of-concept demonstration of the proposed superheterodyne metasurface. For the convenience of off-chip measurement, a Si taper tip, a photonic waveguide, and a planar half-Maxwell fisheye lens are designed to feed the superheterodyne metasurface, as shown in Figure 6(a). The Si taper tip and the photonic waveguide are used to couple energy from the standard WR-1.0 waveguide. Half-Maxwell fisheye lens is a compact gradient index optic that generates a collimated beam fed by a point source at the apex of its circumferential arc, as shown in Figure 6(b). We use the planar half-Maxwell fisheye lens to generate a planar wavefront to feed the superheterodyne metasurface. The design procedure that determines the geometric parameters and hole layout of the Si taper tip, photonic waveguide, and half-Maxwell fisheye lens are described in detail in Supplementary Notes 4 and 5. Both the photonic waveguide and planar half-Maxwell fisheye lens are realized in the form of a 2D lattice of air holes [48, 49]. Therefore, they can be conveniently fabricated together with the superheterodyne metasurface in a single lithography step. Note that the Si taper tip and photonic waveguide are adopted here for latter off-chip measurement purposes, while in practical PICs, photonic circuits can directly feed the superheterodyne metasurface.

We have fabricated a Si-based prototype based on the above design, as shown in Figure 6(c), using deep reactive ion etching (DRIE) and lithography processes. The size of the whole metasurface is 2 mm × 4.42 mm, corresponding to 6.67*λ*_0_ × 14.73*λ*_0_, where *λ*_0_ is the free-space wavelength at 1 THz. The detailed fabrication process is given in the Methods section. Figure 6(d)–(f) show the scanning electron microscope (SEM) images of the fabricated sample. We can observe that the air-hole Si structure is well defined, showing the high accuracy of Si lithography and DRIE technologies. Due to the simple design, our waveguide-integrated metasurface possesses significant advantages of a more straightforward fabrication process and better semiconductor foundry compatibility than previously reported waveguide-integrated metasurfaces with complex meta-atom structures. We experimentally measure the scattering patterns of the metasurface prototype, as shown in Figure 6(g). The signal generator, connected to the extension module, is adopted to launch THz signals. The metasurface is inserted into the WR-1.0 waveguide connected to the receiver. The spectrum analyzer is utilized to record the received power down-converted from the metasurface. A rotary state rotates the metasurface to measure the scattering patterns. The detailed THz off-chip measurement setup is given in the Method section.

The theoretical and measured scattering patterns are illustrated in Figure 6(i)–(k). Limited by the currently available measurement setup, the scattering patterns of the metasurface are measured from 0.95 to 1.05 THz. The measured output angles of the extracted free-space wave at different frequencies are plotted in Figure 6(h). The measured scattering patterns are in good agreement with the theoretical predictions, while some sidelobes are observed for the measured results. The existence of the measured sidelobes is most likely caused by the air gap introduced between the Si metasurface and the gold ground plane in the fabrication process, as shown in Supplementary Figure S7. We can observe from Figure 6(i)–(k) that high-directivity beam can be obtained as an effect of the spatial superheterodyne. We also observe that the output direction of the superheterodyne metasurface steers forward when the operating frequency increases, which matches perfectly with our theoretical model.

## 3 Discussion

A periodic leaky-wave antenna (LWA) consists of a slow-wave transmission line whose structure is periodically modified along the axis of wave propagation [50, 51]. Due to their periodicity, LWAs support an infinite number of space harmonics, including the *n* = −1 space harmonic, which is a fast wave and responsible for radiation. From the perspective of the periodic structure, the superheterodyne metasurface with a sinusoidal baseband signal shown in Section 2.2 can be viewed as a type of periodic LWA. The superheterodyne metasurface’s first lower spatial frequency impulse corresponds to the *n* = −1 space harmonic of LWAs that is radiated into free space. However, the proposed spatial FM metasurface explained from the view of the spatial superheterodyne mechanism provides a clear and insightful understanding of the guided to free-space wave transformation by mimicking the well-known FM/superheterodyne in radio communications. Most importantly, the superheterodyne metasurface is not necessary a periodic structure. Therefore, it enables more flexible free-space light manipulation by judiciously designing the baseband signal. For example, having a baseband signal which is not a sinusoidal wave and a superheterodyne metasurface that is not a periodic structure for focusing applications, as shown in Section 2.4, could not be easily achieved using conventional LWAs.

In the above Theoretical formulation section, uniform amplitude distribution of the waveform is assumed in the superheterodyne metasurface analysis process. The amplitude along the metasurface is spatially changed due to the power progressively coupled from the Si substrate into free space, which is a common phenomenon for the guided to free-space waves transformation devices. One possible solution is to add active amplifiers along the transmission line to regenerate the power progressively coupled into free space, as demonstrated in [52] at microwave frequencies. To investigate the amplitude attenuation effect, the spatial FM waveform in Eq. (3) is modified as

(10) $$E^{\prime }\left(r,t\right)=E_{0}{\text{e}}^{\text{i}\left[\xi_{\text{gw}}x+M\text{sin}\left(\xi_{m}x\right)\right]}{\text{e}}^{-\text{i}\omega t}{\text{e}}^{-\alpha x}u\left(x\right)\delta \left(z\right)\hat{x}$$

where *α* is the attenuation constant related to the power leakage effect, and *u*(*x*) is the Heaviside step function, considering that the waveguide is fed at *x* = 0 and the power is delivered towards +*x* direction. The spatial frequency spectrum of the FM waveform described by Eq. (10) is derived in Supplementary Note 8. The amplitude attenuation effects on the spatial frequency spectrum are given in Figure S8. The space harmonic analysis shows that the attenuation effects do not affect the output angle of the free-space wave. A larger attenuation constant results in a shorter effective waveguide length, thereby a larger beamwidth of the output beam.

## 4 Conclusions

In summary, we have proposed and experimentally demonstrated a superheterodyne-inspired metasurface that bridges the gap between the guided waves inside a waveguide and free-space waves. By simple spatial modulation of the local spatial frequency of the wave in the waveguide for mimicking the FM radio, in-plane guided waves can be transferred to out-of-plane free-space waves. More complex free-space functions, such as 2D arbitrary direction beam deflection and focusing, are also demonstrated. The superheterodyne metasurface is implemented in a Si slab at the THz spectrum as a proof of principle. The technology is easily transposable to near-infrared or visible spectra since they share the same semiconductor processing technology. We choose Si photonics as the demonstration platform here due to its attractive properties, such as low power consumption, high-density integration capability, and complementary metal-oxide-semiconductor (CMOS) compatibility. The methodologies of the superheterodyne metasurfaces can also be equally applied to other integrated photonic material platforms, such as lithium niobate [53–55] or Si on insulator [56, 57]. We have demonstrated that the superheterodyne metasurfaces can achieve momentum compensation. More complex wavefront generations, such as holography [30, 34] are also expected to be implemented by the superheterodyne metasurface by judiciously designing the corresponding baseband signals. For the current superheterodyne metasurface, the functionality is fixed once it is fabricated. The superheterodyne metasurface can be dynamically adjustable by integrating with active materials, such as liquid crystal or graphene with a bias voltage, to change the spatial frequency of the carrier wave.

Our proposed spatial superheterodyne principle provides an entirely new route toward designing advanced waveguide-integrated metasurfaces. The technology empowers integrated photonic devices with extraordinary free-space interactivity capability, enabling various critical applications, especially for the next generation of photonic integrated platforms. Compared to previously reported waveguide-driven metasurfaces [25, 40], [41], [42], [43], [44], [45], our superheterodyne architecture exhibits significant advantages of simpler structure, easier fabrication, and lower loss. Based on the reciprocity principle, the metasurface can also work as a spatial superheterodyne receiver to couple free-space waves into waveguides, which is in high demand for sensing and detection applications. We foresee that the superheterodyne metasurfaces can achieve more complex light manipulation function through the judicious design of baseband signals. Planned future investigations also include the exploration of superheterodyne metasurfaces to manipulate the polarization properties of the extracted free-space light.

## 5 Methods

### 5.1 Sample fabrication

Double side polished Si wafers with high resistivity (*ρ* = 1000 Ω cm), high dielectric constant (*ɛ*_*r*_ = 11.7) and 100 µm thickness were first cleaned and thinned to 40 µm by Si drying etching technology using a deep reactive ion etching (DRIE) system. Then, the 40 µm thick Si wafer was rinsed with acetone, isopropanol, and deionized water for 20 min, respectively, followed by N_2_ drying and dehydrated at 120 °C for 10 min. The Si wafer was then treated with an O_2_ plasma in a reactive ion etching (RIE) system with 20 sccm O_2_, 20 mTorr, and 100 W rf power for 1 min to form a hydrophilic Si surface and coated 1.3 µm thick SPR 6112B resist at 3000 rpm for 1 min. After coating, the resist was prebaked at 95 °C for 5 min and then patterned by optical lithography with ultraviolet exposure for 6 s using SUSS MicroTech MJB4 mask aligner. The SPR6112B resist was then developed for 20 s and hard baked at 95 °C for 10 min. The metasurface pattern was generated on SPR6112B resist, and their residual layer was removed by plasma etching with 20/2 sccm O_2_/SF_6_, 20 mTorr, and 100 W rf power in a RIE system. The SPR6112B metasurface pattern was then used as a mask to etch through the 40 µm thick Si wafer using the DRIE system with the Bosch process for 80 cycles. The Si etching rate and selectivity in this DRIE Bosch process were 0.65 µm/cycle and 108, respectively, with an etch cycle of 120/13 sccm SF_6_/O_2_, 600 W coil power, 14 W platen power, and 30 mTorr for 8 s and a polymer passivation cycle of 85 sccm C_4_F_8_, 600 W coil power, and 16 mTorr for 5 s. The 40 µm thick Si metasurface was etched through with a high aspect ratio, anisotropic profile using the DRIE Bosch process. After Si drying etching, the SPR 6112B resist was removed by plasma etching with 1000 sccm O_2_, 300 W rf power, and 100 mTorr for 20 min. The backside of the Si metasurface was then covered by a polymer sheet and 2/200 nm Cr/Au films were deposited on the tip of the Si metasurface. After Cr/Au deposition, the polymer sheet was removed and the Si metasurface was fixed on a 3D printed holder to help insert the Si tip into the central hole of the WR-1.0 waveguide. Figure S6 summarizes the fabrication processes of the Si metasurface.

### 5.2 Simulation methods

Numerical simulations were carried out using a commercial software package, CST Microwave Studio 2021 (https://www.cst.com/products/cstmws). In the simulation of the local spatial frequency of the Si air-hole structure, eigenmode solver is adopted to find the eigenfrequency of the structure. Periodic boundaries are applied to the boundaries of the unit cell. For a given air-hole radius, the phase delay across the unit cell was swept to find the phase delay corresponding to the operating frequency. Once this phase delay was found, the local spatial frequency could be obtained using *ξ* = *φ*/*l*, where *φ* is the phase delay cross the unit cell at the design frequency of 1 THz, and *l* represents the lattice size of the unit cell. The above simulation process is repeated for each air-hole radius to get the local spatial frequency versus radius design curve in Figure 2(c).

To simulate the beam deflection, a full Si slab model consisting of a Si taper tip, photonic waveguide, half-Maxwell eye lens, and the superheterodyne metasurface was established. The geometric parameters and layout of the air-hole structure are determined based on the design process introduced in the main text. A standard WR-1.0 waveguide with wave port excitation is used to feed the whole Si slab model. Open boundaries are used for all boundaries.

### 5.3 Experimental setups

The diagram and superheterodyne metasurface measurement setup photograph are shown in Figures S3 and S4, respectively. A signal generator (Agilent E8267D) launches a continuous microwave signal. The signal generator is connected to the extension module (VDI SGX 447), in which multiplier chains are deployed to up-convert the frequency to around 1 THz. A diagonal horn (VDI WM-250) is used at the transmitter side to radiate the THz wave into free space. The superheterodyne metasurface works as a receiving device, directly attached to a standard open-ended waveguide (WR-1.0) of the receiver. The mixer in the extension module (VDI SAX 482) and the spectrum analyzer (Agilent E8257D) down-converts the received THz signal to the microwave band. Absorbers are placed around the transmitter and receiver to eliminate possible reflections. Scattering pattern measurement of the superheterodyne metasurface is carried out by rotating the receiver, mounted on a rotary stage, with an angular step of 1°. All the measurement equipment is placed on an optical table with vibration control.

## Supplementary Material

Supplementary Material Details

## References

[1] Sun C., Wade M. T., Lee Y. (2015). Single-chip microprocessor that communicates directly using light. *Nature*.

[2] Feldmann J., Youngblood N., KarpovKarpov M. (2021). Parallel convolutional processing using an integrated photonic tensor core. *Nature*.

[3] Atabaki A. H., Moazeni S., Pavanello F. (2018). Integrating photonics with silicon nanoelectronics for the next generation of systems on a chip. *Nature*.

[4] Papes M., Cheben P., Benedikovic D. (2016). Fiber-chip edge coupler with large mode size for silicon photonic wire waveguides. *Opt. Express*.

[5] Almeida V. R., Panepucci R. R., Lipson M. (2003). Nanotaper for compact mode conversion. *Opt. Lett.*.

[6] Ang T. W., Reed G. T., Vonsovici A., Evans A. G. R., Routley P. R., Josey M. R. (2000). Highly efficient unibond silicon-on-insulator blazed grating couplers. *Appl. Phys. Lett.*.

[7] Orobtchouk R., Layadi A., Gualous H., Pascal D., Koster A., Laval S. (2000). High-efficiency light coupling in a submicrometric silicon-on-insulator waveguide. *Appl. Opt.*.

[8] Roelkens G., Van Thourhout D., Baets R. (2006). High efficiency silicon-on-insulator grating coupler based on a poly-silicon overlay. *Opt. Express*.

[9] Yu N., Genevet P., Kats M. A. (2011). Light propagation with phase discontinuities: generalized laws of reflection and refraction. *Science*.

[10] Ni X., Emani N. K., Kildishev A. V., Boltasseva A., Shalaev V. M. (2012). Broadband light bending with plasmonic nanoantennas. *Science*.

[11] Dai J. Y., Tang W. K., Zhao J. (2019). Wireless communications through a simplified architecture based on time‐domain digital coding metasurface. *Adv. Mater. Tech.*.

[12] Dai J. Y., Zhao J., Cheng Q., Cui T. J. (2018). Independent control of harmonic amplitudes and phases via a time-domain digital coding metasurface. *Light Sci. Appl.*.

[13] Dai J. Y., Tang W. K., Wang M. (2022). Simultaneous in situ direction finding and field manipulation based on space-time-coding digital metasurface. *IEEE Trans. Antenn. Propag.*.

[14] Gao S., Park C. S., Lee S. S., Choi D. Y. (2019). A highly efficient bifunctional dielectric metasurface enabling polarization‐tuned focusing and deflection for visible light. *Adv. Opt. Mater.*.

[15] Chen Z., Deng H., Xiong Q., Liu C. (2018). Phase gradient metasurface with broadband anomalous reflection based on cross-shaped units. *Appl. Phys. A*.

[16] Liu C., Chen L., Wu T. (2019). All-dielectric three-element transmissive Huygens’ metasurface performing anomalous refraction. *Photon. Res.*.

[17] Wang S., Wu P. C., Su V.-C. (2017). Broadband achromatic optical metasurface devices. *Nat. Commun.*.

[18] Khorasaninejad M., Aieta F., Kanhaiya P. (2015). Achromatic metasurface lens at telecommunication wavelengths. *Nano Lett*..

[19] Chen W. T., Zhu A. Y., Sisler J. (2018). Broadband achromatic metasurface-refractive optics. *Nano Lett.*.

[20] Ye W., Zeuner F., Li X. (2016). Spin and wavelength multiplexed nonlinear metasurface holography. *Nat. Commun.*.

[21] Huang L., Zhang S., Zentgraf T. (2018). Metasurface holography: from fundamentals to applications. *Nanophotonics*.

[22] Li Z., Zhang S., Zentgraf T. (2018). Achromatic broadband super‐resolution imaging by super‐oscillatory metasurface. *Laser Photon. Rev.*.

[23] Wan W., Gao J., Yang X. (2017). Metasurface holograms for holographic imaging. *Adv. Opt. Mater.*.

[24] Gao H., Pu M., Li X. (2017). Super-resolution imaging with a bessel lens realized by a geometric metasurface. *Opt. Express*.

[25] Li Z., Kim M.-H., Wang C. (2017). Controlling propagation and coupling of waveguide modes using phase-gradient metasurfaces. *Nat. Nanotechnol.*.

[26] Wang C., Li Z., Kim M.-H. (2017). Metasurface-assisted phase-matching-free second harmonic generation in lithium niobate waveguides. *Nat. Commun.*.

[27] Wang R., Wu Q., Cai W. (2019). Broadband on-chip terahertz asymmetric waveguiding via phase-gradient metasurface. *ACS Photonics*.

[28] Sun S., He Q., Xiao S., Xu Q., Li X., Zhou L. (2012). Gradient-index meta-surfaces as a bridge linking propagating waves and surface waves. *Nat. Mater.*.

[29] Ozaki M., Kato J.-I., Kawata S. (2011). Surface-plasmon holography with white-light illumination. *Science*.

[30] Chen J., Li T., Wang S., Zhu S. (2017). Multiplexed holograms by surface plasmon propagation and polarized scattering. *Nano Lett*..

[31] Zhu H., Xu T., Wang Z. (2018). Flat metasurfaces to collimate electromagnetic waves with high efficiency. *Opt. Express*.

[32] Guan F., Sun S., Xiao S., He Q., Zhou L. (2019). Scatterings from surface plasmons to propagating waves at plasmonic discontinuities. *Sci. Bull.*.

[33] Zhu H., Deng M., Chen S., Chen L. (2019). Graphene-based meta-coupler for direction-controllable emission of surface plasmons. *Opt. Lett.*.

[34] Pan W., Wang Z., Chen Y. (2022). High-efficiency generation of far-field spin-polarized wavefronts via designer surface wave metasurfaces. *Nanophotonics*.

[35] Wang Z., Li S., Zhang X. (2020). Excite spoof surface plasmons with tailored wavefronts using high‐efficiency terahertz metasurfaces. *Adv. Sci.*.

[36] Pors A., Nielsen M. G., Bernardin T., Weeber J.-C., Bozhevolnyi S. I. (2014). Efficient unidirectional polarization-controlled excitation of surface plasmon polaritons. *Light Sci. Appl.*.

[37] Schwartz M., Bennett W. R., Stein S. (1995). *Communication Systems and Techniques*.

[38] Tse D., Viswanath P. (2005). *Fundamentals of Wireless Communication*.

[39] Smith D. R., Yurduseven O., Mancera L. P., Bowen P., Kundtz N. B. (2017). Analysis of a waveguide-fed metasurface antenna. *Phys. Rev. Appl.*.

[40] Guo X., Ding Y., Chen X., Duan Y., Ni X. (2020). Molding free-space light with guided wave–driven metasurfaces. *Sci. Adv.*.

[41] Xie C., Huang L., Liu W. (2021). Bifocal focusing and polarization demultiplexing by a guided wave-driven metasurface. *Opt. Express*.

[42] Guo Y., Pu M., Li X. (2018). Chip-integrated geometric metasurface as a novel platform for directional coupling and polarization sorting by spin–orbit interaction. *IEEE J. Sel. Top. Quantum Electron.*.

[43] Guo R., Decker M., Setzpfandt F. (2017). High–bit rate ultra-compact light routing with mode-selective on-chip nanoantennas. *Sci. Adv.*.

[44] Meng Y., Hu F., Shen Y. (2018). Ultracompact graphene-assisted tunable waveguide couplers with high directivity and mode selectivity. *Sci. Rep.*.

[45] Meng Y., Liu Z., Xie Z. (2020). Versatile on-chip light coupling and (de) multiplexing from arbitrary polarizations to controlled waveguide modes using an integrated dielectric metasurface. *Photon. Res.*.

[46] Memarian M., Eleftheriades G. V. (2015). Dirac leaky-wave antennas for continuous beam scanning from photonic crystals. *Nat. Commun.*.

[47] Hunt J., Driscoll T., Mrozack A. (2013). Metamaterial apertures for computational imaging. *Science*.

[48] Headland D., Fujita M., Nagatsuma T. (2020). Half-maxwell fisheye lens with photonic crystal waveguide for the integration of terahertz optics. *Opt. Express*.

[49] Headland D., Klein A. K., Fujita M., Nagatsuma T. (2021). Dielectric slot-coupled half-maxwell fisheye lens as octave-bandwidth beam expander for terahertz-range applications. ..

[50] Monticone F., Alu A. (2015). Leaky-wave theory, techniques, and applications: from microwaves to visible frequencies. *Proc. IEEE*.

[51] Jackson D. R., Caloz C., Itoh T. (2012). Leaky-wave antennas. *Proc. IEEE*.

[52] Casares-Miranda F. P., Camacho-Peñalosa C., Caloz C. (2006). High-gain active composite right/left-handed leaky-wave antenna. *IEEE Trans. Antenn. Propag.*.

[53] Wang C., Zhang M., Chen X. (2018). Integrated lithium niobate electro-optic modulators operating at cmos-compatible voltages. *Nature*.

[54] Zhang M., Buscaino B., Wang C. (2019). Broadband electro-optic frequency comb generation in a lithium niobate microring resonator. *Nature*.

[55] Wang C., Zhang M., Yu M., Zhu R., Hu H., Loncar M. (2019). Monolithic lithium niobate photonic circuits for kerr frequency comb generation and modulation. *Nat. Commun.*.

[56] Celler G. K., Cristoloveanu S. (2003). Frontiers of silicon-on-insulator. *J. Appl. Phys.*.

[57] Colinge J. P. (2004). *Silicon-on-Insulator Technology: Materials to VLSI*.

