# Supplementary material for: Superheterodyne-inspired waveguide-integrated metasurfaces for flexible free-space light manipulation
Source: Nanophotonics. 2022 Sep 6;11(20):4499–514. doi: 10.1515/nanoph-2022-0352 (PMC11501171; doi:10.1515/nanoph-2022-0352)
Supplement: Supplementary file 1 — Supplementary Material Details [file j_nanoph-2022-0352_suppl.pdf]

Supplementary Materials for

Superheterodyne-Inspired Waveguide-Integrated Metasurfaces for Flexible Free-Space Light Manipulation

Geng-Bo Wu [1,2,†], Shu-Yan Zhu[1,2,3,4,†], Stella W. Pang[1,2,4], and Chi Hou Chan [1,2,*]

[1]*State Key Laboratory of Terahertz and Millimeter Wave, City University of Hong Kong, Hong Kong, 999077, China*

[2]*Department of Electrical Engineering, City University of Hong Kong, Hong Kong, 999077, China*

[3]*School of Microelectronics Science and Technology, Sun Yat-sen University, Zhuhai, 519000, China*

[4]*Centre for Biosystems, Neuroscience, and Nanotechnology, City University of Hong Kong, Hong Kong, 999077, China*

[†]These authors contributed equally to this work.

[*]Corresponding authors. Email: eechic@cityu.edu.hk

This PDF file includes:

Supplementary Notes 1 to 9

Supplementary Figures S1 to S9

Supplementary Table S1

Supplementary Equations S1 to S24

Supplementary Note 1: Derivation of radiation field above the single-tone FM superheterodyne metasurface

The principle of equivalence is adopted to calculate the radiation field above the single-tone FM superheterodyne metasurface. According to the principle of equivalence [1], the equivalent magnetic currents above the metasurface aperture are related to the tangential electric field

$$\begin{aligned}\boldsymbol{M}_s &= -2\hat{n}\times\boldsymbol{E} \\ &= -2E_0 e^{i[\xi_{gw}x+M\sin(\xi_m x)]}e^{-i\omega t}\delta(z)\hat{y}\end{aligned} \tag{S1}$$

The bolded letters represent vectors in Eq. (S1). The magnetic current above will generate EM fields that can be calculated using the electric vector potential $\boldsymbol{F} = \hat{y}F_y$ (*59*). The electric vector potential F_y satisfies the scalar Helmholtz equation

$$\nabla^2 F_y + k_0^2 F_y = -M_s \tag{S2}$$

To obtain F_y, we substitute M_s from Eq. (S1) into Eq. (S2) and expand the equivalent magnetic currents into Floquet series

$$\begin{aligned}\nabla^2 F_y + k_0^2 F_y &= 2E_0 e^{i[\xi_{gw}x+M\sin(\xi_m x)]}e^{-i\omega t}\delta(z) \\ &= 2E_0\sum_{n=-\infty}^{\infty} J_n(M)e^{i(\xi_{gw}+n\xi_m)x}e^{-i\omega t}\delta(z)\end{aligned} \tag{S3}$$

where $J_n(M)$ is the nth order Bessel function of the first kind with the argument M. Inspecting the right-hand side of Eq. (S3), the solution of F_y can be expressed as

$$F_y = \sum_{n=-\infty}^{\infty} A_n(z)e^{i(\xi_{gw}+n\xi_m)x}e^{-i\omega t} \tag{S4}$$

Substituting Eq. (S4) into Eq. (S3), and comparing term by term, we have

$$\frac{\partial^2 A_n(z)}{\partial z^2} + k_{zn}^2 A_n(z) = 0 \tag{S5}$$

for the interested upper half-space $z > 0$. In Eq. (S5), k_{zn} satisfies the following relationship:

$$k_{zn}^2 = k_0^2 - k_{xn}^2 \tag{S6}$$

The solution of Eq. (S5) is

$$A_n(z) = C_n e^{ik_{zn}z} \tag{S7}$$

where C_n is a constant. As a result, F_y can be expressed as

$$F_y = \sum_{n=-\infty}^{\infty} C_n e^{ik_{zn}z}e^{i(\xi_{gw}+n\xi_m)x}e^{-i\omega t} \tag{S8}$$

Using the boundary conditions in z = 0 plane, one can obtain the values of C_n and arrive at the final result of F_y

$$F_y = E_0 \sum_{n=-\infty}^{\infty} \frac{J_n(M)}{ik_{zn}} e^{ik_{zn}z} e^{i(\xi_{gw}+n\xi_m)x} e^{-i\omega t} \tag{S9}$$

The radiated electric and magnetic fields can be found based on the following relationship

$$\boldsymbol{E} = -\nabla \times \boldsymbol{F} \tag{S10}$$

$$\boldsymbol{H} = \frac{\nabla \times \boldsymbol{E}}{i\omega\mu} \tag{S11}$$

The results of nonzero electric and magnetic fields produced by the single-tone FM superheterodyne metasurface are

$$E_x = E_0 \sum_{n=-\infty}^{\infty} J_n(M) e^{ik_{zn}z} e^{i(\xi_{gw}+n\xi_m)x} e^{-i\omega t} \tag{S12}$$

$$E_z = -E_0 \sum_{n=-\infty}^{\infty} \frac{J_n(M)(\xi_{gw}+n\xi_m)}{k_{zn}} e^{ik_{zn}z} e^{i(\xi_{gw}+n\xi_m)x} e^{-i\omega t} \tag{S13}$$

$$H_y = -\omega\varepsilon E_0 \sum_{n=-\infty}^{\infty} \frac{J_n(M)}{k_{zn}} e^{ik_{zn}z} e^{i(\xi_{gw}+n\xi_m)x} e^{-i\omega t} \tag{S14}$$

Supplementary Note 2: Spatial frequency spectrum distributions of baseband, carrier, and FM waveform

The spatial frequency spectrum of the baseband signal is

$$\tilde{F}_{base}(k_x) = \frac{1}{2\pi}\int_{-\infty}^{\infty}\cos(\xi_m x)\, e^{-ik_x x}dx$$
$$= \frac{1}{2}\delta(k_x - \xi_m) + \frac{1}{2}\delta(k_x + \xi_m) \tag{S15}$$

The spatial frequency spectrum of the carrier wave is

$$\tilde{F}_{carrier}(k_x) = \frac{1}{2\pi}\int_{-\infty}^{\infty} e^{i\xi_{gw}x}e^{-ik_x x}dx = \delta\left(k_x - \xi_{gw}\right) \tag{S16}$$

The spatial frequency spectrum of the single-tone FM waveform is

$$\tilde{F}_{FM}(k_x) = \mathcal{F}(e^{i\xi_{gw}x}e^{iMsin(\xi_m x)})$$
$$= \mathcal{F}(e^{i\xi_{gw}x})\otimes\mathcal{F}[e^{iMsin(\xi_m x)}]$$
$$= \mathcal{F}(e^{i\xi_{gw}x})\otimes\mathcal{F}[\sum_{n=-\infty}^{\infty} J_n(M)e^{in\xi_m x}]$$
$$= \delta(k_x - \xi_{gw})\otimes\sum_{n=-\infty}^{\infty} J_n(M)\delta(k_x - n\xi_m)$$
$$= \sum_{n=-\infty}^{\infty} J_n(M)\delta(k_x - \xi_{gw} - n\xi_m) \tag{S17}$$

Supplementary Note 3: Derivation of shift theorem for superheterodyne metasurface

For the baseband signal $m_1(x)$, the FM waveform $E_1(x)$, and its corresponding spatial frequency spectrum $\tilde{F}_1(k_x)$ are

$$E_1(x) = E_0 e^{i\int^x \xi_{gw}+\Delta\xi m_1(x')dx'} = E_0 e^{i\xi_{gw}x} e^{i\int^x \Delta\xi m_1(x')dx'} \tag{S18}$$

$$\tilde{F}(k_x) = E_0\delta(k_x - \xi_{gw})\otimes G(k_x) = E_0 G(k_x - \xi_{gw}) \tag{S19}$$

where $\otimes$ is the convolution operation, and we define the spatial Fourier transform pair $e^{i\int^x \Delta\xi m_1(x')dx'} \xleftrightarrow{FS} G(k_x)$. For the baseband signal $m_2(x) = m_1(x-s)$, using the shift theorem of the Fourier transform, the FM waveform $E_2(x)$ and its corresponding spatial frequency spectrum $\tilde{F}_2(k_x)$ satisfy

$$E_2(x) = E_0 e^{i\int^x \xi_{gw}+\Delta\xi m_2(x')dx'} = E_0 e^{i\int^x \xi_{gw}+\Delta\xi m_1(x'-s)dx'} \tag{S20}$$

$$\begin{aligned}\tilde{F}(k_x) &= E_0\delta(k_x - \xi_{gw})\otimes[e^{-ik_x s}G(k_x)] \\ &= E_0 e^{-i(k_x-\xi_{gw})s} G(k_x - \xi_{gw})\end{aligned} \tag{S21}$$

Compare Eq. (S19) and (S21), one obtains:

$$\tilde{F}_2(k_x) = e^{-i(k_x-\xi_{gw})s}\tilde{F}_1(k_x) \tag{S22}$$

As a result, a space translation of the baseband signal with a distance of s along the y-axis brings an additional phase shift $-(k_x - \xi_{gw})s$.

Supplementary Note 4: Design of Si taper tip and photonic waveguide

A Si taper tip is designed for broadband impedance matching and energy coupling from the standard WR-1.0 waveguide during the off-chip measurement, as shown in Figs. S1a and b. A photonic waveguide based on substrate integrated image guide (SIIG) is subsequently adopted for mechanical stability. The SIIG [2], a kind of substrate integrated circuits, mimics the wave-guiding property of a conventional image guide transmission line. The SIIG consists of a lattice of air holes etched into the Si slab but leaves a core guiding channel in the center. In this manner, the effective permittivity of the guiding core is larger than that of the air-hole region to confine the light within the central guiding core. The lattice periodic of the unit cell is a = 0.04 mm, corresponding to $0.13\lambda_0$. As a result, the structure can be equivalent to an effective bulk homogeneous material. The SIIG operates at a much lower frequency of the photonic bandgap of the periodic hole lattice. The fundamental mode of the SIIG is TM_{11}, and the electric field distribution at 1 THz along the SIIG is shown in Fig. S1c. It is observed that the majority of power is constrained within and propagates along the core Si guide. The specific dimensions of the Si taper tip and SIIG are given in Table S1. Note that the Si taper tip and SIIG are used here for THz off-chip measurement convenience, while the photonic circuits can directly feed the superheterodyne metasurface in practical PICs.

Table S1. Dimensions of the Si taper tip and the SIIG (Unit: μm)

Para.	L_1	L_2	d_1	d_2	w	a	r_1	h
Value	600	100	10	50	80	40	34	40

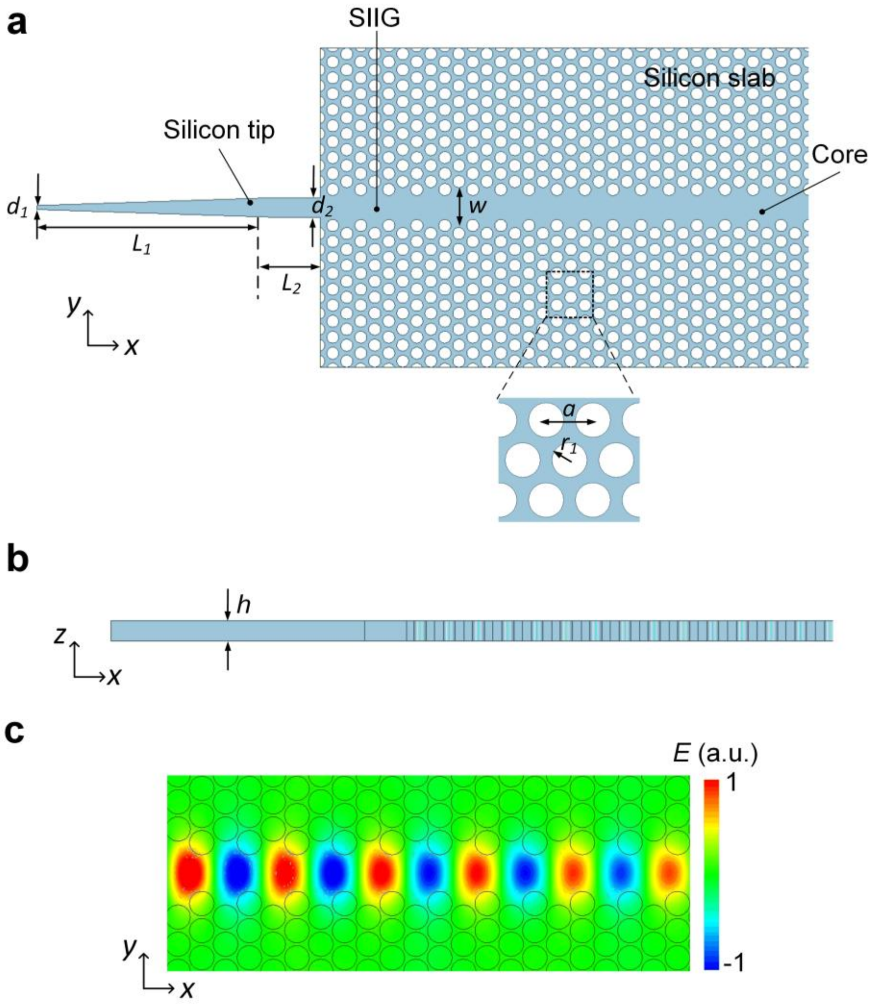

Fig. S1 Design of the Si taper tip and SIIG. (a) Top view. (b) Front view. (c) Numerical simulated electric field distribution of the SIIG in *xy*-plane at 1 THz.

Supplementary Note 5: Design of planar half-Maxwell fisheye lens

A half-Maxwell fisheye lens is a compact gradient index optic [3] that generates a collimated beam fed by a point source at the apex of its circumferential arc, as shown in Fig. S2a. The half-Maxwell fisheye lens is used here to generate a planar wavefront to feed the superheterodyne metasurface. The required refractive index distribution of the Maxwell fisheye lens is

$$n(r) = \frac{n_{\max}}{1 + (\frac{r}{r_{\max}})^2} \tag{S23}$$

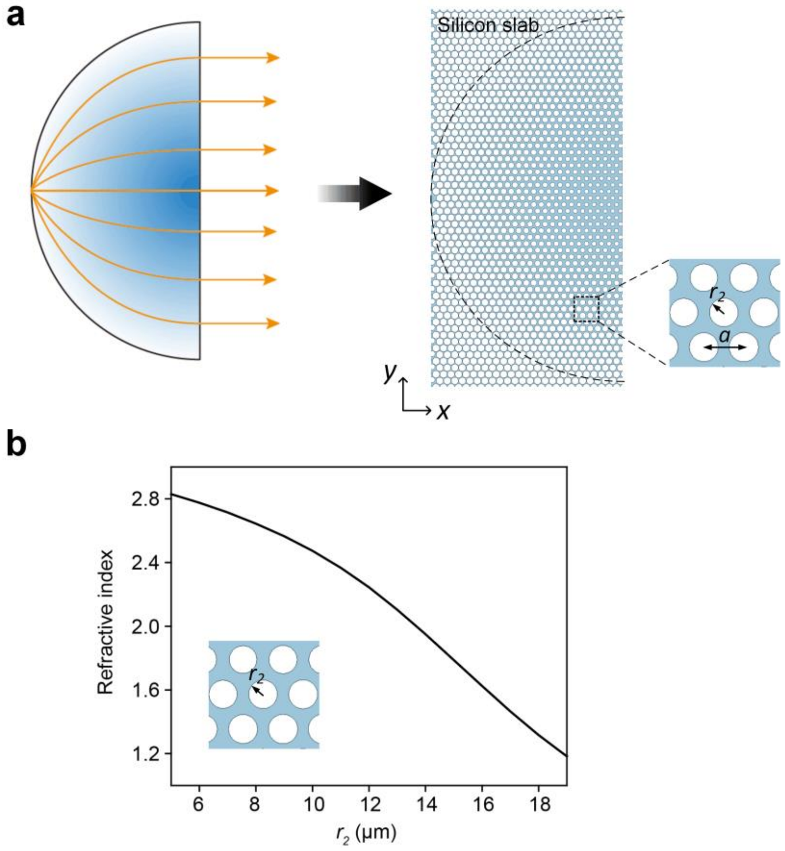

Fig. S2 Design of half-Maxwell fish eye lens based on the Si slab. (a) Operating principle and configuration of the Si-based half-Maxwell fish eye lens. (b) Numerical simulated equivalent refractive index of the air cladding structure as a function of the radius of the air holes at 1 THz.

where r is the radial position of the Maxwell fisheye lens. r_{max} = 1 mm and n_{max} = 2.7 are the radius of the lens and the maximum refractive index located at the center of the lens, respectively. The effective medium at the circumference of the half-Maxwell fisheye lens is

expanded to a rectangular region to increase the mechanical strength. Since the effective refractive index in the expanded region is low (n = 1.2), it does not affect the functionality of the half-Maxwell fish-eye lens. The effective medium is also realized by the 2D lattice of the air holes. The equivalent refractive index of the air cladding structure as a function of the radius of the air hole is given in Fig. S2b, which is obtained by the full-wave simulation using the CST Studio Suite simulator. Subsequently, the corresponding radii of the air cladding of the half-Maxwell fisheye lens can be determined by matching the required refractive indices.

Supplementary Note 6: THz measurement setup

Figs. S3 and S4 show the schematic and photograph of the THz measurement setup for the far-field directional radiation measurement. The fabrication process of the superheterodyne metasurface for focusing is similar to that of the directional radiation. However, the focusing measurement at THz frequencies is much more challenging for our available facilities. In the experiment, the waveguide-fed metasurface should be inserted into a standard WR-1.0 waveguide with a large flange, as shown in Fig. S5(a). To measure the field distribution in Figs. 5(e)-(j), a THz probe should be moved closer to the metasurface in the *xz*-plane to record the radiated fields. Unfortunately, the THz probe available in our lab is bulky, as shown in Fig. S5(b). The translation of the THz probe for focusing measurement will collide with the flange. As a result, only the far-field directional radiation is measured as a proof-of-concept demonstration of the proposed superheterodyne metasurface.

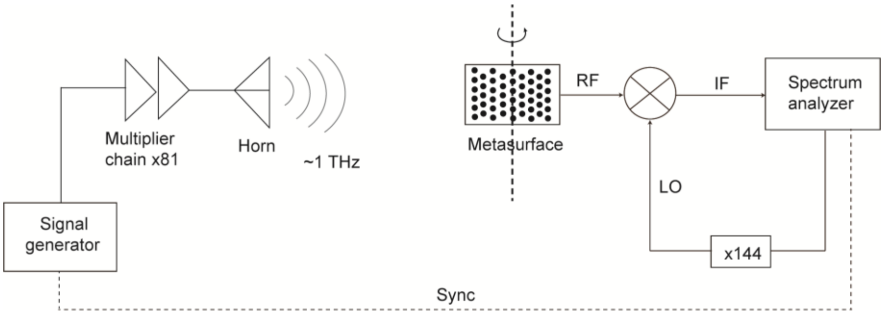

Fig. S3. Block diagram of the THz measurement setup.

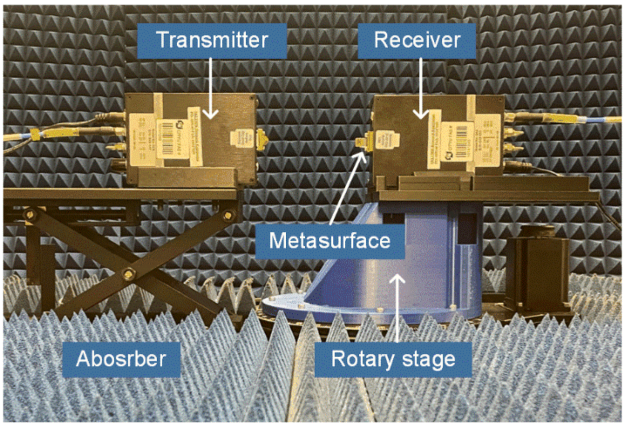

Fig. S4. Photograph of the THz measurement setup for characterizing the scattering pattern of the waveguide-driven metasurface.

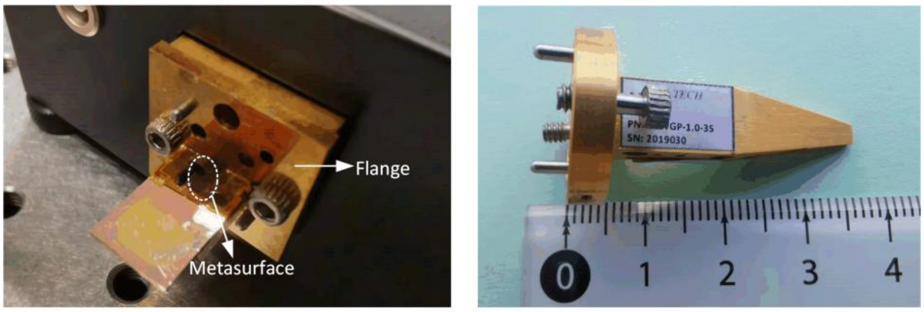

(a) (b)

Fig. S5. (a) Configuration of the waveguide-fed metasurface in the measurement. (b) The available THz probe for focusing measurement.

Supplementary Note 7: Metasurface fabrication processes

Fig. S6 shows the fabrication proceess of the wavegudie-driven metasurface. The existence of the measured sidelobes in Figs. 6(i)-(k) are most likely caused by the air gap introduced between the Si metasurface and the gold ground plane in the fabrication process. Fig. S7 shows the full-wave simulated radiation patterns of the superheterodyne metasurfaces with different air gaps between the Si metasurface and the gold ground plane. Larger sidelobes are generated with the increased air gap. One method to eliminate the air gap is not to etch through the 40 μm thick Si substrate and directly deposit gold on the Si backside in the fabrication process.

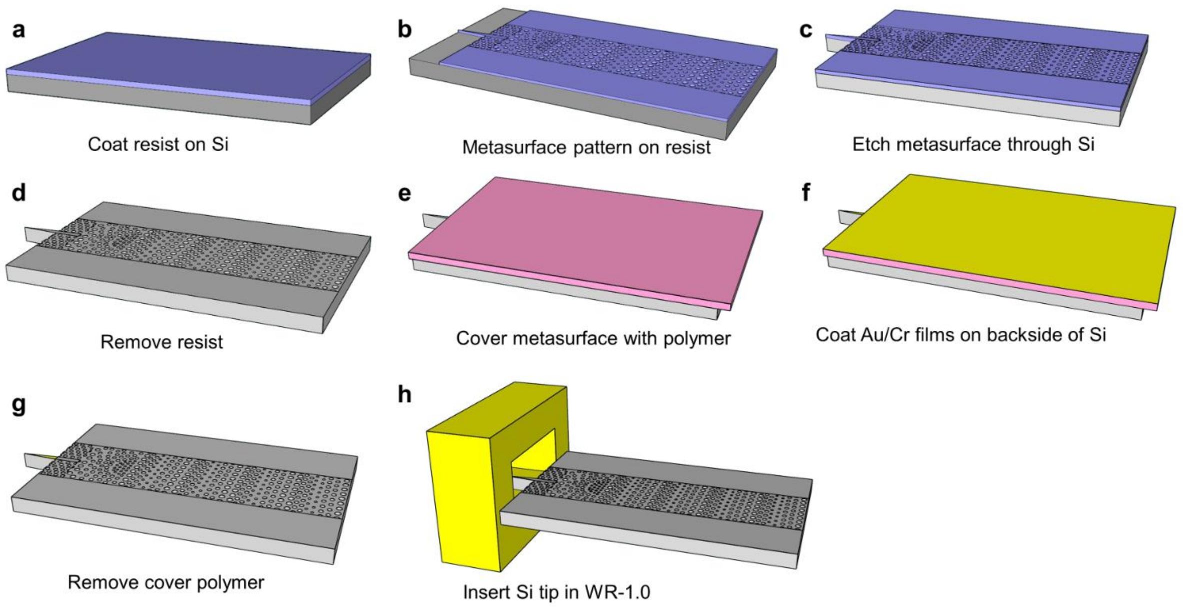

Fig. S6. Fabrication processes of the waveguide-driven metasurface.

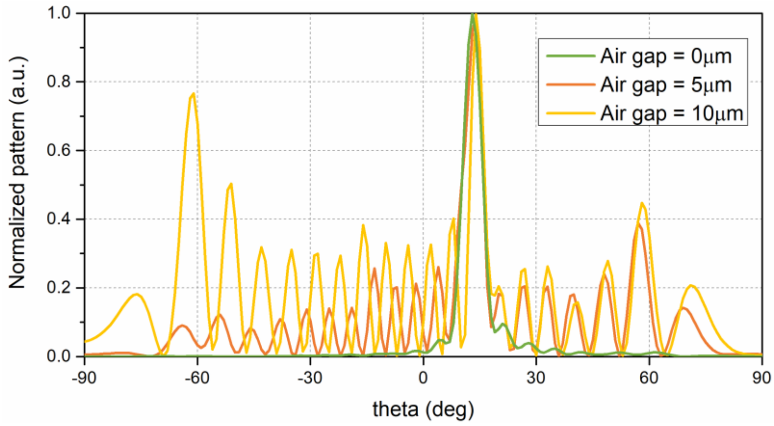

Fig. S7. Simulated normalized radiation pattern of the superheterodyne metasurfaces with different air gaps between the Si metasurface and the gold ground plane.

Supplementary Note 8: Amplitude attenuation effect

The spatial frequency spectrum of the modified spatial FM modulation in Eq. (8) can be calculated using the multiplication property of the Fourier transform

$$\tilde{F}(k_x) = \tilde{F}\{E_0 e^{i[\xi_{gw}x + M\sin(\xi_m x)]} e^{-ax} u(x)\}$$

$$= \tilde{F}\{E_0 e^{i[\xi_{gw}x + M\sin(\xi_m x)]}\} \otimes \tilde{F}\{e^{-ax} u(x)\}$$

$$= E_0 \sum_{n=-\infty}^{\infty} J_n(M)\delta(k_x - \xi_{gw} - n\xi_m) \otimes \frac{1}{2\pi(ik_x + \alpha)}$$

$$= E_0 \sum_{n=-\infty}^{\infty} \frac{J_n(M)}{2\pi[i(k_x - \xi_{gw} - n\xi_m) + \alpha]} \tag{S24}$$

The spatial frequency spectra with different attenuation constants are given in Fig. S8. The spatial spectrum expands for a larger attenuation constant, but the position of the amplitude peak is not affected by the attenuation constant. Therefore, the output angle of the free-space mode is not changed after considering the effects of waveguide amplitude attenuation.

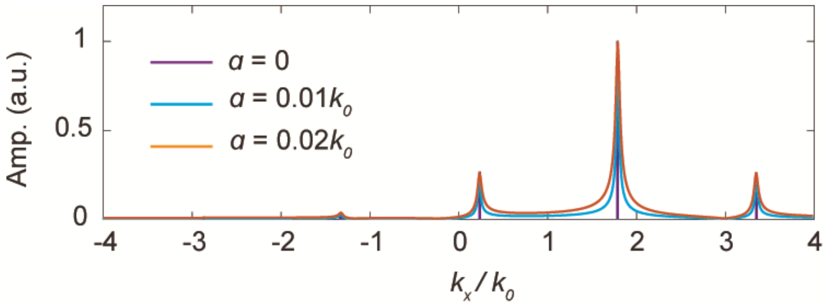

Fig. S8. Spatial frequency distributions of the FM waveforms with different amplitude attenuation constants at 1 THz.

Supplementary Note 9: Truncation effect of the superheterodyne metasurface

To investigate the truncation effect, here we consider superheterodyne metasurfaces with different truncation lengths $L = 6\lambda_0$, $10\lambda_0$, $14\lambda_0$, where λ_0 is the free-space wavelength at 1 THz. Without loss of generality, consider that the spatial frequencies of the unmodulated carrier wave and baseband signal are $1.8\,k_0$ and $1.6\,k_0$, respectively. The calculated normalized radiation patterns of the superheterodyne metasurfaces with different truncation lengths based on Equation (6) are illustrated in Supplementary Fig. S9. It can be observed that sidelobes will be generated due to the truncation of the radiating aperture. Moreover, metasurfaces with a smaller size will have a larger half-power beamwidth.

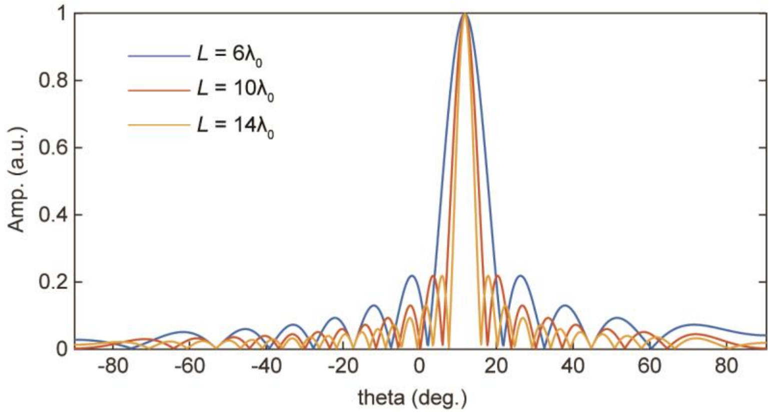

Fig. S9. Calculated normalized radiation patterns of the superheterodyne metasurfaces with different truncation lengths L.

References

[1] A. Lakhtakia, V. K. Varadan, and V. V. Varadan, *Time-harmonic electromagnetic fields in chiral media*: Springer, 1989.

[2] A. Patrovsky, and K. Wu, “Substrate integrated image guide (SIIG)-a planar dielectric waveguide technology for millimeter-wave applications,” *IEEE Trans. Microwave Theory Tech.*, vol. 54, no. 6, pp. 2872-2879, 2006.

[3] B. Fuchs, O. Lafond, S. Palud, L. Le Coq, M. Himdi, M. C. Buck, and S. Rondineau, “Comparative design and analysis of Luneburg and half Maxwell fish-eye lens antennas,” *IEEE Trans. Antennas Propag.*, vol. 56, no. 9, pp. 3058-3062, 2008.
